# Quality of life after ICU: 1-year follow-up in patients with and without COVID

**DOI:** 10.1186/s44158-025-00253-y

**Published:** 2025-07-01

**Authors:** M. Rauseo, G. Ferrara, A. Cotoia, F. Cardinale, S. Padalino, N. Latronico, L. Mirabella, G. Cinnella

**Affiliations:** 1 Anesthesia and Intensive Care Unit, Department of Medical Surgical Science, University Hospital of Foggia, Viale Pinto 1, Foggia, Italy; 2 Oncological Molecular Biology-Clinic Hereditary Familial Tumors, University Hospital of Foggia, Viale Pinto 1, Foggia, Italy; 3 Anesthesia and Critical Care Medicine, Department of Medical and Surgical Specialties, Radiological Science and Public Health, University of Brescia, Brescia, Italy

**Keywords:** Covid-19, Follow-up, PICS, ICU

## Abstract

**Background:**

The purpose of this study was to perform a 1-year follow-up after ICU discharge and evaluate post-intensive care syndrome (PICS) in both COVID (GroupCov) and NON COVID (GroupNCov) patients.

**Methods:**

All consecutive patients discharged from our Intensive Care Unit (ICU) from June to December 2022 were prospectively screened. Scheduled in-person visits were carried on 3, 6, and 12 months after ICU discharge to evaluate physical, cognitive, and mental health status using different scale evaluations (SF-36, Barthel Index, ISI score, PCL-5 score, MNA-sf score, Fatigue Severity Score, MoCA Test, HADS and GDS) by means of standardized questionnaires.

**Results:**

Eighty patients (50 GroupCov vs 30 GroupNCov) were initially included, but some patients did not attend all follow-up visits. At 1-year follow-up, 60 patients (30 COVID-19 and 30 non-COVID) completed all evaluations. Both groups showed PICS, but GroupCov had a better nutritional status, better outcomes in physical evaluations, and a better perception of Quality of Life (QoL) and mental health status, but a worse cognitive assessment in the MoCA Test. Moreover, heterogeneity analysis showed that GroupNCov patients had the same trend during follow-up, while in GroupCov different trends were observed over time, especially a worse nutritional state, often found in older patients, that was related to a longer hospital stay and worse psychophysical outcomes.

**Conclusions:**

This study shows that PICS in SARS-COV2 patients is not always homogeneous, and that different clusters of psychophysical patterns may develop over time.

Although our study was only observational, it seems from our preliminary results that performing a follow-up could be the basis for a secondary prevention and to develop new therapeutic strategies after patients discharge from ICU.

**Supplementary Information:**

The online version contains supplementary material available at 10.1186/s44158-025-00253-y.

## Background

In the last decades, overall patients’ survival after Intensive Care Unit (ICU) dramatically improved, and 28-day and hospital survival rates improved, due to impressive advances in critical care medicine [1–3]. However, during ICU stay, patients do face high levels of psychological stress that could result in cognitive impairments and reduced Quality of Life (QoL) [4]. All these symptoms are incorporated in a syndrome called “post-intensive care syndrome” (PICS) that can occur either during ICU stay or after ICU or hospital discharge [4].

PICS, firstly described by the Society of Critical Care Medicine in 2010, consists of a heterogeneous complex of symptoms characterized by new or increased impairments of physical, cognitive, or psychological functions that outlast the stay in hospital [5]. Cognitive impairments present as deficits in memory, attention, visuospatial perception, or executive functions. Psychological impairments consist of anxiety disorders, insomnia, depression, and post-traumatic stress disorder (PTSD) [6]. Physical impairment consists of ICU-acquired weakness (ICU-AW), one of the most evident results of prolonged ICU stay, due to muscular depletion [7–11]. Since symptoms of PICS in ICU survivors are reported as 25–40% after 3 months and may persist for 5–15 years after discharge [12], numerous authors consider necessary post-ICU follow-up. However, there is still no clear consensus on who should take in charge those patients after ICU stay [13].

PICS may obviously develop also in patients admitted for SARS-COV2 infection [14]. However, in these patients, initial cohort studies such as “PHOS-COVID” [14] evidenced that, besides physical and psychological symptoms, cognitive impairment in these patientsis mainly due to a marked systemic inflammation that activates astrocytes and microglial cells, turning it into a “neuro-Long COVID” (neuro-LC) disease [15].

Long-COVID disease and PICS are thus different entities, but intensivists often are unable to clearly discriminate [14, 16].

Lots of data are present in the literature about PICS in the general ICU population, but only few trials analyzed PICS in COVID-19 patients. Moreover, to our knowledge, there are no studies comparing the incidence of PICS in COVID vs. non-COVID patients admitted to the ICU in the same hospital, in the same period (during the pandemic, with the well-known shortage of personnel and devices) and cared for by physicians and nurses from the same teams, and thus with the same clinical “point of view”).

We therefore launched the present study to evaluate the incidence and the major cause of PICS until 1 year after ICU discharge, in both GroupCov and GroupNCov, and test the hypothesis that in post COVID-19 patients, PICS may be due mainly to a psychological component, since they are often characterized by an optimal quality of life at baseline, with the only impairment due to the pneumonia Sars-COV2 related.

## Materials and methods

### Patients

All consecutive patients discharged from the ICU of “Policlinico Riuniti” University Hospital, in Foggia (Italy), from June to December 2022 were prospectively screened for inclusion criteria, and informed consent was obtained. Inclusion criteria were age ≥ 18 years and length of stay in ICU (ICU-LOS) > 48 h. Patients follow-up starting point was set at 3 months after ICU discharge in order to finalize rehabilitation treatments. Refusal to participate, patients still hospitalized at 90 days after ICU discharge, and patients that had developed the SARS-COV2 infection during the follow-up period were exclusion criteria.

**Fig. 1:**
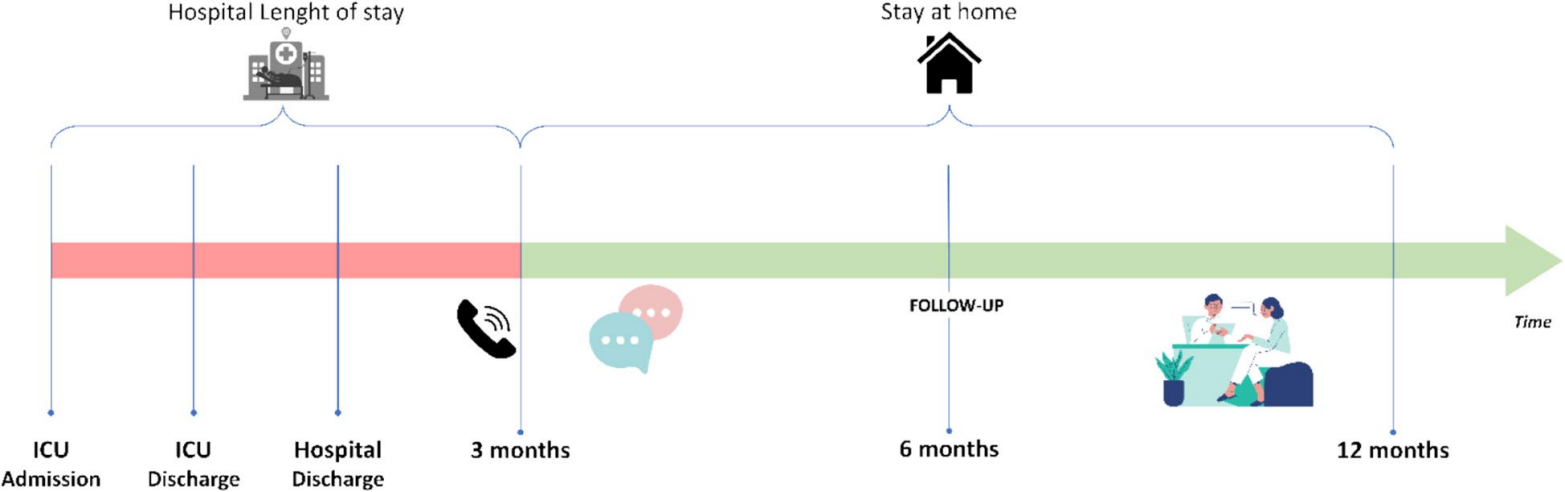
This picture describes the follow-up program that the patient follows over time

This study received approval from the local ethics committee (protocol number 184 of 09/05/2022) and patients satisfying all inclusion criteria were enrolled in a follow-up program involving in-person visits in our outpatient visits at 3, 6, and 12 months after ICU discharge and evaluation via a structured interview (Fig. 1), by a standardized assessment including physical, cognitive, and mental health status, HR quality of life (36-Item Short- Form Health Survey “SF-36”), return to work, and ADL—activity of daily living (via Barthel Index). Cognitive impairments were assessed using the Montreal Cognitive Assessment (MoCA) (Additional file 1).

### Sample size and statistical analysis

The sample size was calculated assuming an expected PICS incidence of approximately 50% in COVID-19 ICU survivors and 35% in non-COVID ICU survivors using the latest data available on the incidence of PICS and PTSD in NON-COVID ICU survivors [1–6, 12, 13] and COVID ICU survivors [14, 16]. To account for an estimated 20–30% loss to follow-up, we prospectively enrolled a larger cohort of 132 patients. Categorical variables are expressed as percentages and continuous variables as M ± DS (mean ± standard deviation) or median (minimum–maximum), as appropriate. Statistical analysis was performed with SPSS version 20 using the Mann–Whitney test.

For repeated measures over time, Bonferroni corrections were applied to minimize type I error due to multiple comparisons. Longitudinal mixed-effects modeling was not performed due to the relatively small sample size and the high dropout rate.

Multivariate analysis (MultiNOVA) was performed between scores with age, sex, and COVID-19 as independent variables. A *p* < 0.05 was considered statistically significant.

## Results

**Fig. 2:**
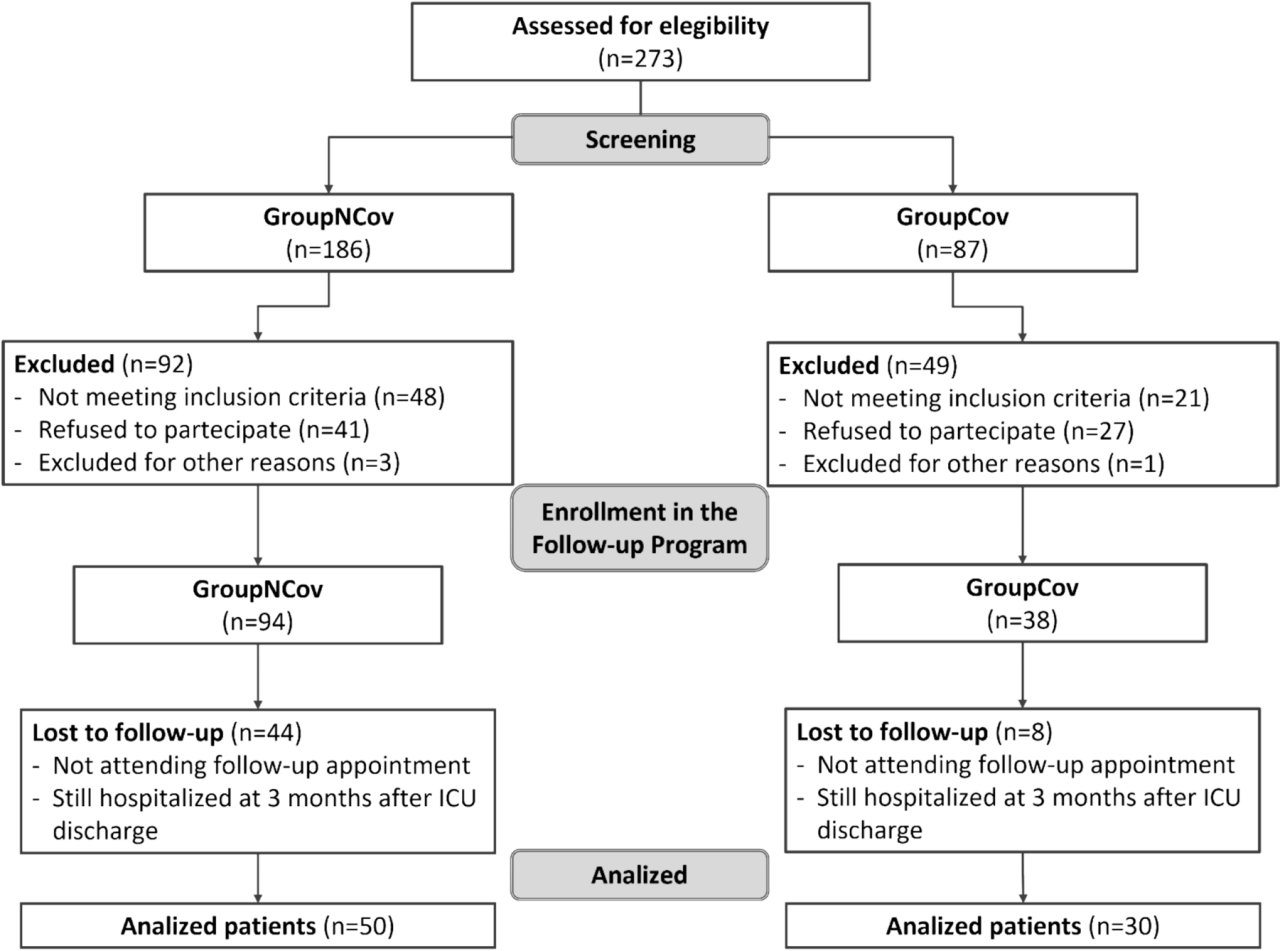
The reported data of the two groups (consort flowchart)

From June to December 2022, 273 patients were assessed for eligibility (87 GroupCov vs 186 GroupNCov) of whom 193 were excluded (49 GroupCov vs 132 GroupNCov). Thus, 132 patients that fulfilled the inclusion criteria were enrolled, and 80 patients (50 GroupNCov and 30 GroupCov) were analyzed (Fig. 2). Causes of ICU admission for patients in the GroupNCov (*n* = 50) were trauma (34%), stroke (20%), acute respiratory distress syndrome (14%), sepsis (14%), voluntary intoxication (8%), hemorrhagic shock (6%), and acute myocardial infarction (4%). Among GroupNCov patients, 36% developed ICU delirium, 28% received neuromuscular blocking agents, and 32% experienced sepsis or septic shock during their ICU stay. The cause of ICU admission for patients in GroupCov (*n* = 30) was acute respiratory distress syndrome (100%). Among these, 40% of patients developed ICU delirium, 30% required the use of neuromuscular blocking agents, and 36% experienced sepsis or septic shock during their ICU stay. Of the 50 non-COVID patients initially enrolled, only 30 completed the entire one-year follow-up program and were included in the final comparison.

In Table 1, demographic data are reported: there were no differences between GroupCov and GroupNCov in terms of age, gender, BMI, physical function in ICU test, SAPS score, duration of ventilation, and length of ICU stay and hospital stay (Table 1).

**Table 1:** Demographic data of the two groups are reported

	**GroupCov (*n* = 30)**	**GroupNCov (*n* = 50)**	***p*-value**
Gender (M:F)	20:10	31:19	> 0.1
Age (years)	61.6 ± 13.1	54.4 ± 16.7	> 0.1
BMI (kg/m^2^)	28.7 ± 5.3	27.1 ± 2.6	> 0.1
Physical function in ICU test	7.2 ± 1.6	7.2 ± 1.8	> 0.1
Length of stay in hospital (days)	40.9 ± 15.6	37.7 ± 18.7	> 0.1
Length of stay in ICU (days)	8.4 ± 4.6	13.4 ± 12.9	> 0.1
SAPS score	41 ± 17	43 ± 14	> 0.1
Duration of ventilation (days)	7 ± 1.7	6.3 ± 1.9	> 0.1

In Table 2, parameters analyzed during follow-up are reported.

**Table 2:** The statistical analysis through median values [Min–Max]

**GroupCov (*n* = 30)**									
	**SF-36**	**Barthel index**	**Fatigue severity**	**Isi score**	**MNA sf**	**PCL 5**	**HADS** (< 65 years)	**GDS** (≥ 65 years)	**MoCA test**
**3 months** (*N* = 30)	37 ^*, #, €^ [25–81]	90 ^*, #, €^ [70–100]	53 ^*, #, €^ [16–62]	11 ^*, #, €^ [0–24]	10 ^*, #, €^ [4–11]	31^*, #, €^ [0–53]	10 ^#, €^ [3–14]	10 ^#, €^ [0–14]	22.5 ^#, €^ [16–26]
**6 months** (*N* = 30)	63 ^*, £^ [29–92]	100 ^*^ [70–100]	42 ^*, £^ [9–54]	6.5 ^£^ [0–18]	11 ^*, £^ [10–14]	20 ^£^ [0–40]	8 ^£^ [0–14]	7 ^£^ 0–11]	25 ^£^ [16–27]
**12 months** (*N* = 30)	71 ^*^ [44–97]	100 ^*^ [80–100]	23 [9–41]	8 [0–19]	12 ^*^ [10–14]	14 ^*^ [0–31]	4 ^*^ [2–10]	5 ^*^ [0–8]	25.5 ^*^ [20–28]
**GroupNCov (*n* = 50)**									
	**SF-36**	**Barthel index**	**Fatigue severity**	**Isi score**	**MNA sf**	**PCL 5**	**HADS** (< 65 years)	**GDS** (≥ 65 years)	**MoCA test**
**3 months** (*N* = 50)	29 ^#, €^ [5–100]	75 ^#, €^ [30–100]	58 ^#, €^ [40–62]	13 ^#, €^ [1–21]	8 ^#, €^ [6–12]	39 ^#, €^ [7–57]	10 ^#, €^ [6–16]	13 ^#, €^ [7–14]	21 ^#, €^ [14–28]
**6 months** (*N* = 35)	57 ^£^ [33–77]	90 [55–100]	40 ^£^ [21–48]	8 ^£^ [0–11]	9 ^£^ [7–14]	24 ^£^ [5–37]	8.5 [1–11]	8 [5–11]	26 ^£^ [20–30]
**12 months** (*N* = 30)	61 [44–80]	90 [70–100]	27 [16–41]	9 [1–15]	10 [9–14]	19.5 [5–31]	8 [1–11]	8 [5–8]	27.5 [23–30]
**MultiNOVA** (Group_Cov_ vs Group_NCov_)	α, β, γ	α, β, γ	α	α, γ	α, β, γ	α, γ	γ	γ	β, γ

Intergroup analysis

* = *p*-value < 0.05 in Mann-Whitney test (GroupCov vs GroupNCov at the same time)

Intragroup analysis

# = *p*-value < 0.05 in Bonferroni test (3 months vs 6 months)

£ = *p*-value < 0.05 in Bonferroni test (6 months vs 12 months)

€ = *p*-value <0.05 in Bonferroni test (3 months vs 12 months)

Multivariate analysis (GroupCov vs GroupNCov *p*-value <0.05)

α = 3 months follow up

β = 6 months follow up

γ = 12 months follow up

*SF-36* Short Form Health Survey 36, *Isi* score Insomnia Severity Index, *PCL5* Post Traumatic Stress Disorder Checklist for DSM-5, *MNA* sf Mini Nutritional Assessment, *MoCA* Montreal Cognitive Assessment, *HADS* Hospital Anxiety and Depression Scale, *GDS* Geriatric Depression Scale

Intragroup analysis shows that the majority of the investigated aspects gradually improved in both groups, reaching the best value at the 1-year follow-up (SF-36; Barthel Index; PCL-5; MNA-sf; Fatigue-severity score; MoCA). The ISI score improved at 6 months in both groups (*p* < 0.05) but slightly worsened at 12 months (*p* < 0.05), although never reaching the 3 months values (*p* < 0.05).

Hospital anxiety and depression scales (HADS and GDS) improved in both groups at 6 months (*p* < 0.05), while at 12 months, they continued to improve in GroupCov (*p* < 0.05) but remained stable in GroupNCov.

Intergroup analysis shows that SF-36, Barthel Index, and MNA-sf value were always better in GroupCov when compared to GroupNCov for the entire follow-up period (*p* < 0.05).

ISI score was always better in GroupCov, but only at 3-month follow-up was there a statistical difference (*p* < 0.05). Pcl-5 score was always better in GroupCov, with significant differences found only at 3 and 12 months (*p* < 0.05). Fatigue Severity Score was better in GroupCov at 3 months (*p* < 0.05); afterward, it also improved in GroupNCov at 6 months (*p* < 0.05), although no differences were found among the two groups at the 1-year follow-up (*p* < 0.05). MoCA Test was always better in GroupNCov, although statistical differences among the two groups are present only at 12 months (*p* < 0.05). HADS and GDS showed a difference between the two groups only at the 1-year follow-up (*p* < 0.05). The observed prevalence of PICS symptoms at 1 year in both groups (particularly fatigue, cognitive decline, and PTSD features) was consistent with the predicted incidence used for our sample size calculation (approximately 50% in GroupCov and 35% in GroupNCov).

Finally, in order to exclude bias related to demographic characteristics, we performed a MultiNOVA analysis that showed differences found in our results were related to COVID-19.

**Fig. 3:**
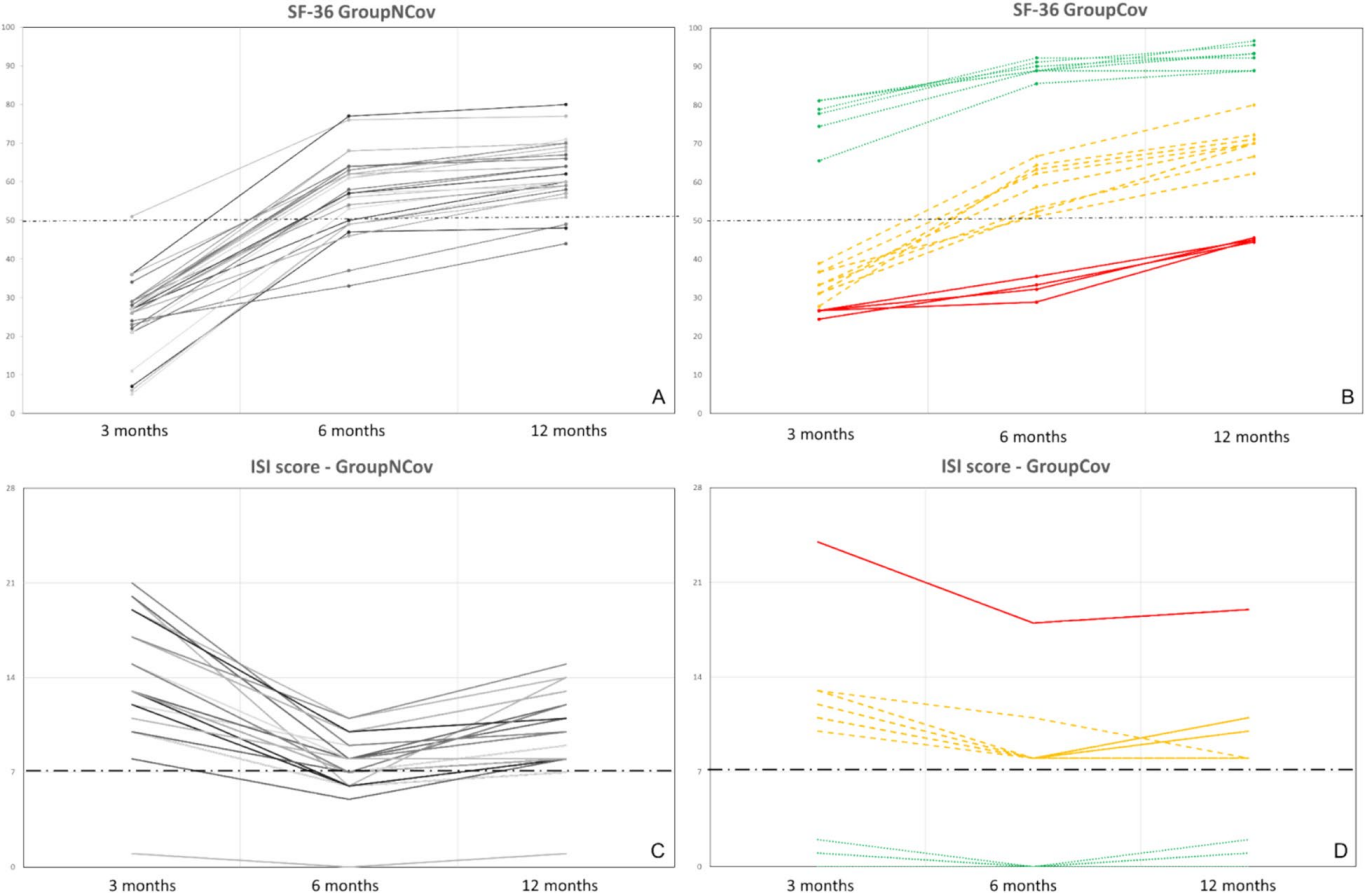
Individual analysis of data during follow-up. **A** GroupCov “SF-36 scale”, **B** GroupNCov “SF-36 scale”, **C** GroupCov “ISI score”, **D** GroupNCov “ISI score”

We also analyzed SF-36 and ISI score time course in individual patients: for both scores in Group NCov (Fig. 3A and C), a similar trend over time was observed in every patient during the whole follow-up period, while in GroupCov (Fig. 3B and D), two different behaviors were evidenced:

- Twelve patients (40%) had at 3 months an SF-36 > 50 and an ISI score < 7 (i.e., values showing absence of insomnia) (Fig. 3C, D). Both scores improved at 6 months (*p* = 0.01) and remained stable at 12 months (*p* = 0.11) (Fig. 3B and D). These patients were middle-aged patients (59.2 ± 12.6 years) with a short H-length of stay (31.8 ± 13.1 days) and a low level of malnutrition (MNA-sf scale) and chronic fatigue (Fatigue Severity scale);
- The remaining 18 patients (60%) had at 3 months an SF-36 < 50 (32.5 ± 5.3) and an ISI score > 7. With the exception of one outlier (patient n. 24 that suffered from chronic insomnia), all had ISI between 8 and 14 (i.e., values showing sub-threshold insomnia) at 3 months. ISI score remained unchanged during the whole follow-up, while:
  - **✓ **In 14 out of these 18 patients, SF-36 increased over 50 at 6 months (58.1 ± 5.8; *p* < 0.01), and at 12 months (69.8 ± 4.3; *p* < 0.01), these patients were also middle-aged (59.3 ± 10.1 years), but they had a longer H-length of stay (42 ± 11.9 days), presented mild PTSD symptoms (PCL-5 scale) and mild cognitive decline (MoCA Test), and were characterized by a mild level of malnutrition (MNA-sf scale) and mild chronic fatigue (Fatigue Severity scale);
  - **✓ **In the remaining 4 patients, SF-36 remained below the threshold at 6 months (33.7 ± 1.69; *p* = 0.06) and at 12 months it improved only slightly but remained < 50 (46.7 ± 2.9; *p* = 0.17): these 4 patients were older (77 ± 16.2 years) with even longer H-length of stay (64.5 ± 4.8 days). Although this subgroup presented mild PTSD symptoms (PCL-5 scale) and mild cognitive decline (MoCA Test), they were characterized by a serious level of malnutrition (MNA-sf scale) and serious chronic fatigue (Fatigue Severity scale).

Interestingly, all differences in SF-36 scale data are related in every patient only to 3 subscales: SF-36-RP (role-functioning physical), SF-36-RE (role-functioning emotional). and SF-36-GH (general health).

## Discussion

The major results of our study are as follows:

1. Patients admitted to ICU because of SARS-COV2-related pneumonia (GroupCov) hada less accentuated PICS with better outcomes when compared to patients admitted to ICU for other causes during the whole period, especially with regard to the physical evaluation (Barthel index score), most likely related to a better nutritional status (MNA-sf scale) and this, obviously, translates into a better perception of quality of life (SF-36scale) and a supposed lower perception of psychic compromise (PTSD scale, HADS scale, and GDS scale).
2. Nonetheless, COVID-19 patients showed a worse cognitive outcome (MoCA scale).
3. No differences between the two groups in some simple but fundamental aspects of daily life, such as sleep quality (ISI score) or chronic fatigue perception (Fatigue Severity Score), were observed.
4. Interestingly, the perception of quality of life during the follow-up after hospital discharge did not influence GroupNCov. In contrast, further analysis on GroupCov showed different behaviors over time, for which an older age is associated with a longer hospital stay and thus, to a worse psychophysical outcome.

To better understand the complexity of our findings, a brief discussion on PICS is needed.

As already stated, PICS is characterized by physical, cognitive, or psychological functions’ impairment, which may express heterogeneously in individual patients or in specific cohorts [17]. Cognitive and psychological dysfunctions real incidence is probably underestimated: Geense and coworkers showed that the incidence of frailty, fatigue, muscle weakness, anxiety, depression, and cognitive impairment at 1 year post-ICU recovery is more frequent than expected [18], and it has been confirmed that patients with delirium in the ICU are at a greater risk of long-term outcomes of cognitive dysfunction [12, 19]. Moreover, patients admitted to the ICU experience environmental stimuli, particularly noise and light [20], so improving sleep quality by using noise reduction devices was suggested as helpful to reduce the development of delirium.

The novelty of the present study, in our opinion, is represented firstly by the contemporary evaluation of PICS incidence in both COVID and NON COVID patients, admitted at the same time, treated by the same team of physicians, in the same institution, with the same equipment and protocols, that made no bias related to different working groups in different working contexts, during a worldwide pandemic with a lack of personnel. Moreover, strictly from our data analysis, we noticed how, although the psychological component of ICU is often underestimated, quality of sleep (ISI score) worsened at 12 months, likely related to the anniversary of the tragic event, as many patients stated, thus confirming the importance of both physical and psychological aspects.

Xie and colleagues [21] evaluated mental health outcomes in patients with COVID and found an increased risk of anxiety disorders, depressive disorders, and stress and adjustment disorders at 30 days after COVID compared with contemporary uninfected controls. In our study, we have no differences between the 2 groups (MoCA score) at 3 and 6 months (*p* > 0.05), whereas we have differences at 12 months (*p* = 0.00004) with worse outcomes in GroupCov, thus confirming previous data.

An important cause of PICS is represented by a major reduction in the physical activity of daily life (ADL) post-ICU discharge, generally worse than ICU admission, that can reach the loss of autonomy in some cases. The effects of physical therapy-based post-ICU follow-up on quality of life in five studies [22] for a total of 348 patients showed no differences between the intervention and control groups regarding the physical component score of quality of life. In our study, GroupCov has always had a better perception of quality of life (SF-36 score), although an objective analysis with the Barthel index score shows persistent differences between the two groups.

Nutritional therapy is vital for the prevention of PICS, especially ICU-AW. Previous studies on nutrition therapy targeted mortality and infectious complications as outcomes. With the recent opinion that nutrition therapy should target muscle volume and strength [23], there is a strong connection between nutritional therapy and PICS. Although studies have shown that the securement of minimum energy delivery with supplemental parenteral nutrition from the acute phase was associated with decreased PICS, overfeeding could induce autophagy impairment and worsen ICU-AW. Therefore, clinicians should select the appropriate energy delivery and avoid overfeeding [24]. In our study, all patients were nourished according to the ESPEN guidelines, but GroupCov had a better nutritional outcome (MNA-sf score) during the whole observation period. Adequate protein delivery with total energy could reduce PICS; however, a number of studies have reported that protein delivery alone does not reduce PICS. As for the particular type of nutrition, leucine is the amino acid reported to induce muscle protein synthesis. Unfortunately, administration of specific amino acids including leucine did not show efficacy in critically ill patients. GroupCov, despite nutritional appropriate protocols, presents persistent neurological deficits lasting for months or years after acute COVID, as has already been reported since the early stages of the pandemic. Many patients with neurological symptoms meet the diagnostic criteria for having symptoms consistent with ME/CFS (myalgic encephalomyelitis/chronic fatigue syndrome) [25]. ME/CFS is a chronic, multisystem disease affecting about 20 million people worldwide that manifests as chronic fatigue, post-exertional malaise, and cognitive impairment. In our study (Fatigue Severity Score) GroupCov has better values during the entire period.

Critically ill patients with COVID might have been uniquely affected by social isolation resulting from restricted visitation in most hospitals during the pandemic. In addition, various cross-sectional comparative studies report that 37% of critically ill patients over 65 years of age in the ICU had pre-existing cognitive impairment [26] and ICU stay could get it even worse. Depression arises in approximately 30% of ICU survivors, anxiety in 70%, and PTSD in 10–50% of them [27]. In our study, there were no differences between the two groups in patients younger than 65 years old (HADS score) in follow-up at 3 months and 6 months, whereas we found differences at 12 months (*p* = 0.02) with better value in GroupCov. Moreover, in patients older than 65 years old (GDS score), there were no differences in follow-up at 3 months and 6 months, whereas we have differences at 12 months (*p* = 0.02) with better value in GroupCov. Interestingly, none of our patients were prescribed with antidepressants drugs.

This study has some limitations: (1) luckily, the pandemic outbreak ended since the vaccination campaign began, so we could not enroll more COVID-19 patients; (2) despite we treated numerous patients, most of them refused to participate in all meetings; (3) baseline functional status and quality of life data before ICU admission were not available, which limits our ability to fully contextualize the patients’ recovery trajectories after ICU discharge; and (4) most importantly, we have not yet standardized a method to match ICU discharge to hospital discharge, thus our “month count” does not always correspond, resulting in a bias that needs to be addressed for further follow-ups. Another major limitation of our study is that it does not compare COVID-19-related respiratory failure with non-COVID respiratory failure alone, but rather with a heterogeneous group of ICU admissions including trauma, stroke, and sepsis. Future studies should investigate more homogeneous populations to better isolate the impact of different etiologies on PICS development.

## Conclusion

In conclusion, our data show that taking care of critically ill patients involves, among the auspicial outcomes, the most appropriate treatments to resume their previous quality of life or, at least, the assumed one for a person of similar age and medical condition.

SARS-COV2 pandemic exacerbated that ICU stay modulates not only physical aspects, but also cognitive and mental ones: these aspects must be investigated during ICU stay and followed over time. Although the concept is not a novel, new diseases like COVID-19 show that different pathologies need different approaches to improve patients’ outcomes, accounting for longer follow-ups. Patients must be cured and followed from all points of view to feed the most important thing for them after ICU discharge: a concrete hope for their future.

## Supplementary Information

Supplementary Material 1

## Data Availability

No datasets were generated or analysed during the current study.

## Abbreviations

PICS: Post-Intensive Care Syndrome
ICU: Intensive Care Unit
GroupCov: COVID group
GroupNCov: Non COVID Group
SF-36: Short Form Health Survey-36
ISI: Insomnia Severity Index
PCL-5: Post-traumatic Stress Disorder Checklist
MNA-sf: Mini Nutritional Assessment short-form
MoCA: Montreal Cognitive Assessment
HADS: Anxiety and Depression Scale
GDS: Geriatric Depression Scale
QoL: Quality of life
ICU-AW: Intensive care unit-acquired weakness
PTSD: Post-traumatic stress disorder
LOS: Length of stay
H-LOS: Hospital length of stay
ARDS: Acute respiratory distress syndrome
ADL: Activity of daily living
