# Supplementary material for: Quality of life after ICU: 1-year follow-up in patients with and without COVID
Source: J Anesth Analg Crit Care. 2025 Jul 1;5:36. doi: 10.1186/s44158-025-00253-y (PMC12211204; doi:10.1186/s44158-025-00253-y)
Supplement: Supplementary file 1 — Supplementary Material 1 [file 44158_2025_253_MOESM1_ESM.pdf]

SUPPLEMENTARY INFORMATION 1

SF-36

The SF-36 is a brief yet comprehensive measure of general health status that consists of eight scales yielding two summary measures: physical and mental health. The physical health measure includes four scales of physical functioning (10 items), role-physical (4 items), bodily pain (2 items), and general health (5 items).

The mental health measure is composed of vitality (4 items), social functioning (2 items), role-emotional (3 items), and mental health (5 items). A final item, termed self-reported health transition, is answered by the patient but is not included in the scoring process. Likert scales and yes/no options are used to assess function and well-being on this 36-item questionnaire. Scoring the SF-36, scales are standardized with a scoring algorithm or by the SF-36v2 scoring software to obtain a score ranging from 0 to 100. Higher scores indicate better health status; a score of 50 has been articulated as a mean one.

Barthel index

The Barthel Index assesses an individual's ability to perform 10 basic activities of daily living. It was employed to assess their functional capacity. This scale provides a numerical assessment of the patient's reliance, graded from 0 to 100, where higher scores signify greater self-sufficiency. The Barthel Index is classified into four categories: 80-100 (Fully independent, with a score of 100 representing complete self-reliance); 60-79 (Minimally dependent); 40-59 (Moderately dependent); and <20 (Completely dependent).

Fatigue severity scale

Additionally, we assessed fatigue levels using the Fatigue Severity Scale (FSS), which comprises nine items probing how fatigue impacts an individual's activities and everyday life over the preceding two weeks [24]. Each item comprises statements rated on a 7-point Likert-type scale, ranging from 1 ("strongly disagree") to 7 ("strongly agree"). The average score of these items serves as the FSS score, where a higher score indicates more pronounced fatigue severity. A score of FSS ≥36 is deemed indicative of severe fatigue. The minimum score = 9 and maximum score possible = 63. Higher the score = greater fatigue severity.

ISI score

The Insomnia Severity Index (ISI) is a self-reported questionnaire consisting of 7 items designed to assess the nature, severity, and impact of insomnia. It evaluates various dimensions such as the seriousness of difficulties falling asleep, staying asleep, and experiencing early morning awakening, dissatisfaction with sleep, interference of sleep problems with daytime functioning, how noticeable sleep issues are to others, and the distress caused by sleep difficulties. Each item is rated on a 5-point Likert scale (e.g., 0 = no issue; 4 = extremely severe issue), resulting in a total score ranging from 0 to 28. The total score is interpreted as follows: absence of insomnia (0-7); sub-threshold insomnia (8-14); moderate insomnia (15-21); and severe insomnia (22-28).

MNA-sf

The Mini Nutritional Assessment short-form (MNA-SF) is classified as: normal nutritional state (MNA-SF score: 12-14); risk for malnutrition (8-11); malnourished (0-7).

PCL-5

The PCL-5 is 20 items measure of the 20 DSM-5 symptoms of Post-Traumatic Stress Disorder (PTSD). Included in the scale are four domains consistent with the four criteria of PTSD in DSM-5:

- *Re-experiencing (criterion B);*
- *Avoidance (criterion C);*
- *Negative alterations in cognition and mood (criterion D);*
- *Hyper-arousal (criterion E).*

The PCL-5 can be used to monitor symptom change, to screen for PTSD, or to make a provisional PTSD diagnosis.
Scores consist of a total symptom severity score (from 0 to 80) and scores for four subscales:

- *Re-experiencing (items 1-5 – max score = 20);*
- *Avoidance (items 6-7 – max score = 8);*
- *Negative alterations in cognition and mood (items 8-14 – max score = 28);*
- *Hyper-arousal (items 15-20 – max score = 24).*

Consistent with the Likert scale: 0 = Not at all; 1 = A little bit; 2 = Moderately; 3 = Quite a bit; 4 = Extremely. A cut-off score is 38 for a provisional diagnosis of PTSD. This cut-off has high sensitivity (.78) and specificity (.98).If the scale is used to track symptoms over time, a minimum 10 point change represents clinically significant change.

Hospital anxiety and depression scale

This brief self-report measure is specifically designed for individuals dealing with somatic comorbidities. It comprises two subscales, each consisting of seven items that assess anxiety and depression separately. Each of the 14 items presents a statement reflecting a particular mood state, followed by four options indicating the frequency of experiencing that feeling in the past week. Scores on each subscale range from 0 to 21.
Scores between 8 and 10 suggest the likelihood of clinically significant depression/anxiety, scores above 11 indicate the need for a formal diagnosis, and scores exceeding 15 suggest a severe affective disorder. The Hospital Anxiety and Depression Scale (HADS) is regularly used by ICU teams due to its ease of use for both evaluators and respondents. It exhibits good internal reliability, construct validity, discriminative and predictive validity, is well-received by patients in acute settings, and has been validated for use within intensive care populations.

Geriatric depression scale

The Geriatric Depression Scale (GDS) is a self-report tool used to measure depression in older adults. Answers are given in a simple yes/no format. Originally, the GDS was a 30-item instrument, but due to its time-consuming and difficult nature for some patients, a 15-item version was developed. The shortened form, known as GDS-S, consists of 15 items selected from the Geriatric Depression Scale-Long Form (GDS-L). These 15 items were selected due to their strong correlation with depressive symptoms in previous validation studies. Of these 15 items, 10 indicate the presence of depression when answered positively, while the remaining 5 indicate the presence of depression when answered negatively. The questionnaire can be completed in approximately 5 to 7 minutes, making it ideal for individuals who experience fatigue or have limited ability to concentrate for extended periods of time. Scores ranging from 0 to 4 are considered normal, depending on age, education, and complaints. Scores between 5 and 8 indicate mild depression, while scores between 9 and 11 indicate moderate depression. Scores between 12 and 15 indicate severe depression.

MoCA – Montreal cognitive assessment

MoCA test, a concise cognitive screening tool endorsed by the Society of Critical Care Medicine for ICU patients, was administered in person. MoCA evaluates different cognitive aspects including visuospatial and executive functioning (5 points), animal naming (3 points), attention (6 points), language (3 points), abstraction (2 points), delayed recall (short-term memory, 5 points), and orientation (6 points). The maximum achievable score is 30, indicating optimal cognitive performance. A suggested cutoff score for normal cognitive function is 26/30. If patients score below 26, we utilized the following categorization: 18- 25 = mild cognitive impairment, 10-17 = moderate cognitive impairment, and below 10 = severe cognitive impairment.
